# Comparison of the fecal microbiota of adult healthy dogs fed a plant-based (vegan) or an animal-based diet

**DOI:** 10.3389/fmicb.2024.1367493

**Published:** 2024-04-17

**Authors:** Brooklynn D. Liversidge, Diego E. Gomez, Sarah A. S. Dodd, Jennifer L. MacNicol, Jennifer L. Adolphe, Shauna L. Blois, Adronie Verbrugghe

**Affiliations:** ^1^Department of Clinical Studies, Ontario Veterinary College, University of Guelph, Guelph, ON, Canada; ^2^Department of Population Medicine, Ontario Veterinary College, University of Guelph, Guelph, ON, Canada; ^3^Department of Animal Biosciences, Ontario Agricultural College, University of Guelph, Guelph, ON, Canada; ^4^Department of Veterinary Biomedical Sciences, Western College of Veterinary Medicine, University of Saskatchewan, Saskatoon, SK, Canada; ^5^Petcurean Pet Nutrition, Chilliwack, BC, Canada

**Keywords:** canine, alternative diets, companion animal nutrition, microbiome, plant-based ingredients, vegan

## Abstract

**Purpose:**

Pet guardians are increasingly seeking vegan dog foods. However, research on the impact of these diets on gastrointestinal (GI) physiology and health is limited. In humans, vegan diets modify the GI microbiota, increasing beneficial digestive microorganisms. This study aimed to examine the canine fecal microbiota in response to a vegan diet compared to an animal-based diet.

**Methods:**

Sixty-one client-owned healthy adult dogs completed a randomized, double-blinded longitudinal study. Dogs were randomly assigned into two groups that were fed either a commercial extruded animal-based diet (MEAT, *n* = 30) or an experimental extruded vegan diet (PLANT, *n* = 31) for 12 weeks. Fecal collections occurred at the start of the experimental period and after 3 months of exclusively feeding either diet. Bacterial DNA was extracted from the feces, and the V4 region of the 16S rRNA gene was amplified using PCR and sequenced on Illumina MiSeq. Beta-diversity was measured using Jaccard and Bray–Curtis distances, and the PERMANOVA was used to assess for differences in fecal microbiota within and between groups. Alpha-diversity indices for richness, evenness, and diversity, as well as relative abundance, were calculated and compared between groups.

**Results:**

Beta-diversity differences occurred between diet groups at exit time-point with differences on Bray–Curtis distances at the family and genus levels (*p* = 0.007 and *p* = 0.001, respectively), and for the Jaccard distance at the family and genus level (*p* = 0.006 and *p* = 0.011, respectively). Significant differences in alpha-diversity occurred when comparing the PLANT to the MEAT group at the exit time-point with the PLANT group having a lower evenness (*p* = 0.012), but no significant differences in richness (*p* = 0.188), or diversity (*p* = 0.06). At exit-timepoint, compared to the MEAT group, the relative abundance of *Fusobacterium*, *Bacteroides*, and *Campylobacter* was lower in the PLANT group. The relative abundance of *Fusobacterium* decreased over time in the PLANT group, while no change was observed in the MEAT group.

**Conclusion:**

These results indicate that vegan diets may change the canine gut microbiota. Future studies are warranted to confirm our results and determine long-term effects of vegan diets on the canine gut microbiome.

## Introduction

To most pet guardians, dogs are viewed as members of the family. This has resulted in pet nutrition trends to closely following popular trends in human nutrition (Buff et al., 2014; Knight and Leitsberger, 2016; Dodd et al., 2018). Consumption of an entirely plant-based (vegan) diet that avoids consuming any ingredients that come from an animal has increased in popularity in human nutrition because of the potential positive impacts on the environment and overall health (Berschneider, 2002; Michel, 2006; Dodd et al., 2018; Eisen and Brown, 2022; Feigin et al., 2023; Knight, 2023). As more pet guardians switch to a vegan diet, the potential for moral conflicts has developed when it comes to feeding their pets because most commercial diets are formulated using animal-based ingredients (Rothgerber, 2013; Dodd et al., 2018). A survey conducted in 2019, indicated that three quarters of vegan pet guardians would reportedly feed a plant-based diet to their pets if one was available to meet their needs (Dodd et al., 2019). A third of pet guardians who did not already feed a plant-based diet expressed interest in doing so, and a fifth of those pet guardians who expressed interest stated that further knowledge about plant-based diets is needed before they will feed such diet to their pets (Dodd et al., 2019). The gap in required research to determine the feasibility of vegan diets for dogs pertains to the long-term nutritional suitability with regards to the ability of these diets to meet all nutritional requirements (Berschneider, 2002; Michel, 2006; Remillard, 2008; Dodd et al., 2018, 2019; Knight et al., 2022).

The limited research regarding nutritional adequacy of vegan diets, poses questions for both veterinarians and pet guardians on their long-term sustainability. Commercial vegan diets for dogs have been voiced by veterinary nutritionists as potentially being insufficient in meeting the maintenance requirements stated by the National Research Council, The Association of American Feed Control Officials (2019), and the European Pet Food Industry Federation (McMillan et al., 2006; Beloshapka et al., 2016; Dodd et al., 2021). These concerns are based on lower concentrations of nutrients and poor bioavailability of nutrients in plant-based ingredients used in vegan diet formulations (Dodd et al., 2021). Digestibility studies however, on individual plant ingredients, mildly cooked human-grade, and whole plant-based diets have demonstrated that plant-based ingredients if processed correctly have the potential to demonstrate similar nutrient digestibility when compared to conventional animal-based ingredients (Kendall and Holme, 1982; Bednar et al., 2000; Liversidge et al., 2023). This is likely due to coevolution of dogs and humans which has led to dogs becoming closer related biologically to humans rather than to their wolf ancestors (Buff et al., 2014). Unlike their wolf ancestors, the canine gut microbiota shows to have greater ability to adapt to either a high-protein or high-carbohydrate diet and contains a diverse number of bacteria used for fermentation of these nutrients (Alessandri et al., 2019). Specifically, the gut microbiota of dogs has adapted to show an increase in gut microbiota used for fermentation of plant material and reduction of gut microbiota used in the fermentation of protein and fats from animal ingredients (Coelho et al., 2018; Alessandri et al., 2019; Liu et al., 2021). Thus, if a vegan diet that was formulated with highly digestible plant-ingredients was consumed, the canine gut microbiota has the ability to shift making it possible for dogs to meet daily nutrient requirements with consumption of a plant-based diet.

In humans, diet has a large impact on the richness and diversity of microorganisms present within the gastrointestinal (GI) tract (Bosch et al., 2009; Kamboj and Nanda, 2018; Alvarenga et al., 2019). Individuals following a vegan diet compared to an omnivorous diet showed differences in the diversity and abundance of several taxa of the GI microbiota (Honda and Littman, 2012; Singh et al., 2017). Specifically, the gut microbiota has an increased abundance of bacterial communities that aid in fermentation and absorption of plant-materials and a decreased abundance of communities that breakdown animal-derived protein and fat sources (Winglee and Fodor, 2015; Zhenjiang and Knight, 2015; Singh et al., 2017). Vegetarian – devote of meat products but including dairy and eggs – or vegan diet consumption has been seen to shift gut microbiota in a state of dysbiosis to aid in reducing inflammation and treating conditions such as inflammatory bowel disease, obesity, and type-2 diabetes in humans (Li et al., 2020; Santhiravel et al., 2022; Sahoo et al., 2023). This shift in the GI microbiota appears to be helpful to properly digest and utilize plant-based ingredients and is essential for plant-based diets to provide beneficial effects. Some researchers have investigated fecal microbial effects of single plant-based ingredients in dogs (Carciofi et al., 2010; Bazolli et al., 2015; Hankel et al., 2020); however, currently, there is no research conducted on the effects of vegan diets on the gut microbiota in dogs.

Following thousands of years of domestication, the GI microbiota of dogs and humans have developed structural and functional similarities (Coelho et al., 2018). Based on these resemblances, we hypothesized that similar alterations in microbial taxonomy seen in humans consuming a vegan diet would occur in dogs fed a diet consisting of only plant-based ingredients, in combination with mineral or synthetic additives, but devote of meat-based ingredients. Therefore, the objective of this study was to examine the canine fecal microbial composition and structure in response to an experimental vegan diet compared to a commercially available animal-based diet.

## Materials and methods

All experimental procedures for this study were approved by the University of Guelph Animal Care Committee (AUP#4192) and the Research Ethics Board (Research Ethics Approval Number 19-02-036), and were in accordance with institutional, provincial, and national guidelines for the care and use of animals and humans participating in research.

### Animals and experimental design

This study was conducted as part of a larger randomized, double-blinded longitudinal study in client-owned healthy adult dogs, which occurred between July 2019 and November 2020 (Dodd et al., 2023). Recruitment of trial participants was conducted through an eSurvey designed on the Qualtrics (Provo, UT, USA) platform to collect data regarding suitability for study enrollment. Details regarding this recruitment survey were published previously (Dodd et al., 2023; Liversidge et al., 2023). The survey was advertised locally around the University of Guelph campus and surrounding community, as well as shared virtually on social media to local dog-related groups. Dogs were excluded from enrollment consideration if they were reproductively intact, had a body weight (BW) less than 5 kg, had an owner reported body condition scores (BCS) greater than 5 (WSAVA, 2013), were fed homemade or raw pet food, were housed outdoor without supervision, had access to unmonitored food sources, had current medical problems, or had any known dietary allergies. Dogs in households without children or other animals were prioritized. Recruitment resulted in a total of 87 dogs scheduled for enrollment appointments (Supplementary Figure 1).

The enrollment appointment included discussion of the study procedures, collection of a signed informed consent from participants and a wellness examination of the dogs conducted by a licensed veterinarian. The wellness examination included a medical and dietary history, physical examination, and a bodyweight measurement. Blood was collected for complete blood count and serum biochemistry. Dogs were approved for inclusion in the trial if they were confirmed to be spayed/neutered, aged 3 years or older, had a BCS between 4 and 7 on a 9-point scale (Laflamme, 1997), and were deemed healthy based on a physical examination and routine blood work. Seventy-six dogs met the inclusion criteria and started the 4-week adaptation period during which all dogs received the same commercial extruded animal-based diet (MEAT) (Supplementary Figure 1). Eleven dogs did not continue the study after the adaptation period due to not eating the diet, GI abnormalities, excessive weight gain, or COVID-related pet guardian dropouts.

The remaining 65 dogs were randomly assigned into two diet groups; continuing with the animal-based diet (control group; MEAT, *n =* 31) or being fed an experimental extruded vegan diet (PLANT, *n =* 34) (Supplementary Figure 1). Diets were fed for 12 weeks, maintaining current energy intake, as determined based on diet history information. Four dogs were excluded during the experimental period (MEAT, *n =* 1; PLANT, *n =* 3) due to pet guardian personal reasons or dog health concerns unrelated to diet including development of GI ulcers after administrations of non-steroidal anti-inflammatory drugs and development of a urinary tract infection. Fecal collections occurred at the end of the 4-week adaptation period (baseline time-point) and after 3 months of exclusively feeding either the MEAT or PLANT diet (exit time-point). Owners were instructed to collect fecal samples immediately after voiding and as close to their appointment as possible, freeze and deliver the samples to the research team in a provided container stored in a Styrofoam cooler box. Upon arrival, fecal samples were immediately stored frozen at −20°C until the microbiome analyses could be completed.

Due to the COVID-19 pandemic, the trial was paused for 4 months from March 2020 until July 2020. During this period dogs were maintained on the experimental diets, either PLANT or MEAT depending on which phase of the trial they were in (adaptation or feeding trial) to allow for immediate resumption of data collection when restrictions were lifted. This resulted in some variation in trial duration for dogs participating in the study, with the adaptation period for five dogs lasting more than 4 weeks (PLANT, *n* = 2; MEAT, *n* = 3), the experimental period for two dogs lasting more than 12 weeks (PLANT, *n =* 1; MEAT, *n =* 1), and for three dogs both the adaptation period and the experimental period lasting more than 4 and 12 weeks, respectively (PLANT, *n =* 2; MEAT, *n =* 1). During this period, frequent communication between the research team members and the pet guardians was maintained. This was performed to aid as a reminder to stay consistent with trial protocol during the extended 4 months.

### Diets

Both diets, MEAT and PLANT, used in this study were formulated to meet or exceed nutrient recommendations according to The Association of American Feed Control Officials (2019) nutrient profile for canine adult maintenance. The MEAT diet was a commercial extruded dog food (Petcurean Go! Solutions Skin + Coat Care Chicken Recipe, PPN Ltd., Chilliwack, BC, Canada). The PLANT diet was an experimental extruded dog food, excluding all animal-based ingredients, formulated to be isoenergetic and as similar as possible in macronutrient and micronutrient profiles to the MEAT diet (Supplementary Table 1). Proximate analysis and mineral analysis were performed on both diets post-manufacturing at a commercial laboratory (Bureau Veritas, Mississauga, ON, Canada). Details regarding the diets used were published previously (Dodd et al., 2023; Liversidge et al., 2023). Pet guardians and researchers were blinded to the identity of the diets being fed to study participants throughout the duration of the testing period and remained blinded until after all data were analyzed.

Food quantity was calculated based on the dogs’ current dietary intake to match calories and maintain current BW. A gram scale was provided to each household to precisely measure the recommended quantity of food per day. Pet guardians were given a list of plant-based treats without added micronutrients. An acceptable treat dose was calculated for each dog to avoid exceeding 10% of their daily energy intake from sources other than the experimental PLANT or MEAT diets. Pet guardians were instructed not to feed their dogs any other food items as well as to record food and treat intake in a daily food diary for the duration of the study.

### Sample preparation and DNA extractions

Whole fecal samples were thawed in their original containers in a refrigerator (4°C) the night before DNA extractions. Bacterial DNA was extracted using a commercial stool extraction kit (E.Z.N.Z Stool DNA Kit, Omega Bio-Tek Inc., Doraville, GA, USA) according to the manufacturer’s instructions. Extracted DNA samples were stored in a −80°C freezer until further analysis.

### Polymerase chain reaction

To test the quantity of extracted DNA a spectrophotometer (NanaDrop 1000 Spectrophotometer, NanoDrop Technologies Inc., Thermo Fisher Scientific, Waltham, MA, USA) was used on all extracted DNA samples. The DNA extractions were thawed in rounds of 27 samples and diluted to a range of 30–100 ng/ml. The V4 region of the 16S rRNA gene was amplified using polymerase chain reaction (PCR) with the forward primer 515F(5′GTGCCAGCMGCCGCGGTAA-3′) and reverse primer 806R(5′GGACTCTACHVGGGTWTCTAAT-3′) (Walters et al., 2016), KAPA HiFi ReadyMix (Kapa Biosystems, Wilmington, MA, USA), and PCR grade water. The PCR products were purified with Mag Bind RXNPure plus (Omega Biotek Inc.). To prepare the purified PCR products for Illumina MiSEq sequencing, the purified PCR products were amplified using Illumina adapters N716-N729 and S513-S522, and then purified again. All finalized PCR products were evaluated using gel electrophoresis and DNA was measured using spectrophotometry to ensure the concentration of DNA was greater than 15 ng/μl before Illumina sequencing.

### DNA sequencing

Bridge amplification was completed on an Illumina MISeq system (Illumina, San Diego, CA, USA) at the University of Guelph Agri-Food Labs using terminator nucleotides that were incorporated into the amplified PCR products with the removal of the terminator group (Tal et al., 2020; Chui et al., 2023).

### Sequence processing

Following DNA sequencing of fecal samples, Mothur v1.48.0 was used for sequence processing according to the software standard operating procedures (Schloss et al., 2009; Kozich et al., 2013). Assembly of paired end reads was performed using the make.contigs command to extract the sequences and create its reverse complement to join the reads into contigs. Next multiple filtrations were conducted using a variety of screen.seqs commands to remove sequences greater than 250 base pairs (bp) in length and those with any ambiguous base calls or runs of homopolymers greater than 8 bp. Alignment of sequences to the Silva v132 16S rRNA reference database was completed, with removal of sequences that did not align with the correct region (Quast et al., 2013).With the use of chimera.vsearch command all identified chimeras were removed to further remove sequencing errors. Sequences were clustered into operational taxonomic units using 97% sequences similarity and taxonomy assigned using the Ribosomal Database Project classifier (v14) (Cole et al., 2014).

### Statistical analysis

To examine community membership and structure, Beta diversity indexes were assessed using the Jaccard and Bray–Curtis distances and PERMANOVA tests were used to assess for the effect of time, diet, and its interaction on both distances in R (R Core Team, Vienna, Austria). Visual similarities and clustering of each diet was plotted with principal coordinate analysis (PCoA) using the package ggplot2. Evenness [how even the abundance of species is within a community (Hagery et al., 2020)], richness [total number of different species present in the community (Hagery et al., 2020)] and diversity (evenness and richness) were calculated using Shannon diversity (Shannon, 1948), and Chao (Chao, 1984) and Simpson diversity (Simpson, 1949) indices, respectively. Normality of the data distribution was assessed using the Shapiro–Wilk test. The Wilcoxon tests were used to compare alpha-diversity indexes between diet groups (PLANT and MEAT) and between time-points (baseline and exit). Relative abundance (%) was calculated for the different taxonomic levels for each diet. The relative abundance was calculated and comparison between and within groups was conducted among phyla with a relative abundance >1% (10 most abundant) phyla, >0.01% for family, and >0.001% for genus. Differences were evaluated using nonparametric Wilcoxon tests, with *p*-values adjusted for multiple comparisons (Benjamini and Hochberg, 1995). Relative abundances are presented as median with range (minimum and maximum). A *p <* 0.05 for all comparisons was considered statistically significant. Labeled scatter plots were created for each significantly different dependent variable to identify potential outliers.

## Results

Of the total 61 client owned dogs completing the study, 47 were included for fecal microbiota analysis (27 consuming PLANT and 20 consuming MEAT). Sample size was reduced due to a lack of fecal samples submitted by the pet owners on the day of sample collection (*n =* 5), poor DNA yield after extraction (*n =* 7), or a repeated inability to obtain adequate sequence numbers (*n =* 2).

As mentioned previously, the COVID-19 pandemic added variation in trial duration for 10 dogs participating in the study. Examination of labeled scatter plots revealed these dogs were not identified as outliers and results were not significantly different from the dogs consuming the experimental diet for the intended 12 weeks.

The dogs were aged 3 years or older (mean age being 4.734 ± 0.347), both neutered male (*n =* 22) and spayed female (*n =* 25) of various purebred or crossbreeds. All dogs enrolled in this study had BCS between 4 and 7 on a 9-point-scale with BW ranging between 5 and 50 kg (baseline BW: 24.12 ± 1.48; exit BW: 24.13 ± 1.53). All dogs in the study tolerated their diets well, with none showing signs of malnutrition or illness during the baseline or trial periods.

### Fecal microbiota analysis

Fecal analyses resulted in a total of approximately 14,673,953 sequences. Following quality control filtering a total of 11,725,298 sequences remained, with a median of 117,591 sequences per sample (range: 88,435–209,427). To standardize sequence numbers used for analysis, subsampling was completed based on the smallest number of sequences from a sample. The coverage was then assessed using Good’s coverage value.

### Beta-diversity

**FIGURE 1:**
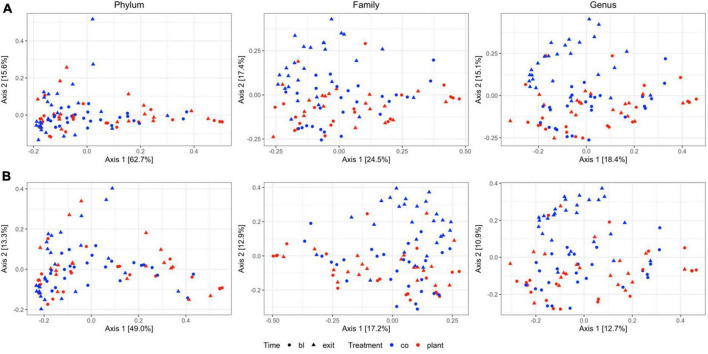
Two-dimensional principal coordinate analyses plots of **(A)** community structure (Bray–Curtis distances) and **(B)** community membership (Jaccard distances) of 47 healthy adult client-owned dogs fed an experimental plant-based (PLANT, *n* = 27) or commercial animal-based (MEAT, *n* = 20) extruded diet in a 12-week randomized, double-blinded longitudinal study. Samples are colored by treatment (CO, MEAT diet; plant, PLANT diet), and time-points are shaped by time (circle, baseline; triangle, exit). Figure panels in each column are grouped to the taxonomic level.

Comparison between diet groups at entrance time-point revealed no significant differences on Bray–Curtis and Jaccard distance at the phylum, family, or genus levels. Similarly, in both the PLANT and MEAT groups over time (baseline – exit), no significant differences in Bray–Curtis and Jaccard distance at the phylum, family, or genus levels were found. Comparison between diet groups at the exit time-point revealed differences on Bray–Curtis distances at the family and genus level (*p =* 0.007 and *p =* 0.001, respectively) (Supplementary Table 2) and for the Jaccard distance (*p =* 0.006 and *p =* 0.011, respectively) (Supplementary Table 3) but differences at the phylum level were not identified (Supplementary Tables 2, 3). Principal coordinate analyses plots obtained from Jaccard and Bray–Curtis distances showed no apparent clustering of samples based on diet groups and time-points (Figure 1).

### Alpha-diversity indices

Alpha-diversity indices for richness, evenness, and diversity between diet group and time-point are described in Table 1. There were no differences in alpha-diversity indices when comparing the PLANT to MEAT group at baseline. Comparing the baseline to the exit time-point of the MEAT group, MEAT had greater richness (Chao index, *p =* 0.028) but not evenness (*p =* 0.839) or diversity (*p* = 0.081) at the exit time-point. Similarly, when comparing the baseline to exit time-points of the PLANT group, the exit samples had lower evenness than the baseline samples (*p =* 0.036) but no differences in richness (*p =* 0.387) or diversity index (*p* = 0.368). When comparing the PLANT to the MEAT group at the exit time-point, the PLANT group had lower evenness (*p* = 0.012) but no differences in richness (*p =* 0.188) or diversity (*p =* 0.060).

**TABLE 1:** Alpha-diversity indices (Chao, Richness; Shannon, evenness; and Inverse Simpson’s, diversity) observed in the feces of 47 healthy adult client-owned dogs fed an experimental plant-based (PLANT, *n* = 27) or commercial animal-based (MEAT, *n* = 20) extruded diet in a 12-week randomized, double-blinded longitudinal study.

Alpha-diversity Indexes	Diet group and time-point		*p*-Value
Chao: richness	PLANT^1^ baseline	MEAT^2^ baseline	0.57
	460.53 [264.75–1,254.56]	427.33 [201.26–854.91]	
	PLANT^1^ exit	MEAT^2^ exit	0.12
	488.81 [295.23–1,288.89]	616.14 [342.52–1,498.64]	
	PLANT^1^ baseline	PLANT^1^ exit	0.39
	460.53 [264.75–1,254.56]	488.81 [295.23–1,288.89]	
	MEAT^2^ baseline	MEAT^2^ exit	0.03^a^
	427.33 [201.26–854.91]	616.14 [342.52–1,498.64]	
Shannon: evenness	PLANT^1^ baseline	MEAT^2^ baseline	0.80
	0.54 [0.34–0.63]	0.51 [0.38–0.62]	
	PLANT^1^ exit	MEAT^2^ exit	0.01^a^
	0.47 [0.28–0.61]	0.53 [0.36–0.60]	
	PLANT^1^ baseline	PLANT^1^ exit	0.04^a^
	0.54 [0.34–0.63]	0.47 [0.28–0.61]	
	MEAT^2^ baseline	MEAT^2^ exit	0.84
	0.51 [0.38–0.62]	0.53 [0.36–0.60]	
Inverse Simpson’s: diversity	PLANT^1^ baseline	MEAT^2^ baseline	0.45
	11.20 [2.73–22.67]	9.13 [3.12–19.88]	
	PLANT^1^ exit	MEAT^2^ exit	0.06
	8.74 [2.48–19.66]	12.98 [3.41–24.91]	
	PLANT^1^ baseline	PLANT^1^ exit	0.37
	11.20 [2.73–22.67]	8.74 [2.48–19.66]	
	MEAT^2^ baseline	MEAT^2^ exit	0.08
	9.13 [3.12–19.88]	12.98 [3.41–24.91]	

Comparisons were made between the baseline and exit time-points for each diet group. As data was presented as non-parametric alpha-diversity indices between diet group at each timepoint are presented as median and interquartile range [minimum and maximum].

^1^PLANT, plant-based diet.

^2^MEAT, animal-based diet.

^a^Coefficient of correlation significant at *p <* 0.05.

### Relative abundance

**FIGURE 2:**
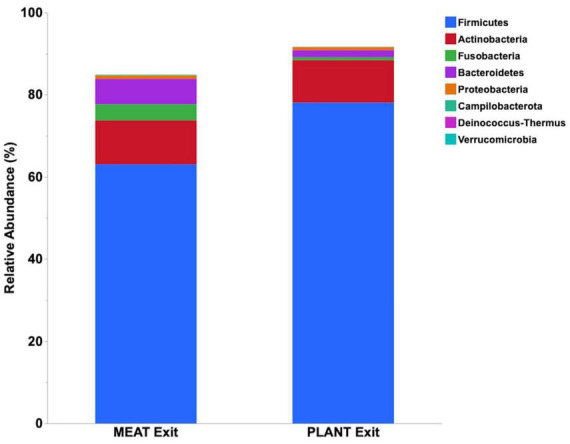
Comparison of median relative abundance of the main phyla identified in feces 47 healthy adult client-owned dogs fed an experimental plant-based (PLANT, *n* = 27) or commercial animal-based (MEAT, *n* = 20) extruded diet in a 12-week randomized, double-blinded longitudinal study. Comparisons were made after the dogs were exclusively fed either the experimental PLANT diet or commercial MEAT diet for 3 months.

**FIGURE 3:**
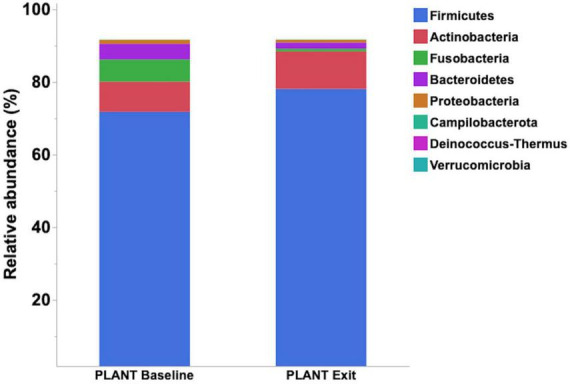
Comparison of median relative abundance of the main phyla identified in feces of 47 healthy adult client-owned dogs fed an experimental plant-based (PLANT, *n* = 27) or commercial animal-based (MEAT, *n* = 20) extruded diet in a 12-week randomized, double-blinded longitudinal study. Comparisons were made after the dogs were exclusively fed either the experimental PLANT diet or commercial MEAT diet for 3 months.

Median relative abundance was examined across all taxa between diet groups and time-points. After performing a Benjamini–Hochberg adjustment, significant changes in relative bacterial abundance between diet groups and time were present at the phylum level (Table 2); however, almost all changes to the family and genus level were no longer present (Supplementary Tables 4, 5, respectively). In the MEAT group over time, on the family level only Streptococcaceae (*p* = 0.02) was significant and on the genus level only Bifidobacterium (*p* = 0.03) and Streptococcus (*p* = 0.03) was significant (Supplementary Tables 4, 5, respectively). The most abundant phyla in both diet groups were Firmicutes, Actinobacteria, Fusobacteria, and Bacteroidetes. The most abundant families in both diet groups were Peptostreptococcaceae, Lachnospiraceae, Fusobacteriaceae, and Erysipelotrichaceae. The most abundant genera in both diet groups were Peptacetobacter, Blautia, Fusobacterium, and Collinsella. The relative abundance of three of the predominant phyla differed between diet groups at the exit time-point, with Firmicute*s* being higher in the dogs fed the PLANT diet (*p* = 0.021), while Fusobacteria, Bacteroidetes, and Campilobacterota were lower in dogs fed the PLANT diet (*p =* 0.013, *p =* 0.012, and *p =* 0.001, respectively) (Figure 2). The relative abundance of Fusobacteria (*p <* 0.001) decreased from baseline to exit (*p =* 0.001) in dogs fed the PLANT diet (Figure 3). No differences were observed overtime in the MEAT diet group. No other differences were noted in relative abundance across all other taxa between diet groups and time-points.

**TABLE 2:** Median relative abundance of predominant taxonomic classifications on the phylum level of bacteria from the feces of 47 healthy adult client-owned dogs fed an experimental plant-based (PLANT, *n* = 27) or commercial animal-based (MEAT, *n* = 20) extruded diet in a 12-week randomized, double-blinded longitudinal study.

Phylum	PLANT^1^ baseline	MEAT^2^ baseline	*p*-Value	FDR^3^
Firmicutes	71.77 [34.16–91.01]	70.31 [27.40–91.92]	0.94	0.94
Actinobacteria	8.33 [0.65–15.97]	6.06 [0.54–23.99]	0.27	0.80
Fusobacteria	6.11 [0.27–60.63]	6.73 [0.21–32.09]	0.91	0.94
Bacteroidetes	4.31 [0.23–20.60]	6.87 [0.14–39.08]	0.40	0.84
Proteobacteria	1.06 [0.44–36.22]	1.77 [0.02–15.45]	0.67	0.87
Campilobacterota	0.04 [0–4.58]	0.10 [5e–^4^–2.69]	0.15	0.69
Deinococcus-Thermus	0 [0–0.01]	0 [0–0.01]	0.55	0.84
Cyanobacteria/Chloroplast	0.01 [9e^–4^–0.48]	6.2e^–3^ [0–0.06]	0.09	0.69
Verrucomicrobia	2.7e^–3^ [0–0.08]	2e^–3^ [0–0.07]	0.56	0.84
**Phylum**	**PLANT^1^ exit**	**MEAT^2^ exit**	***p*-Value**	**FDR^3^**
Firmicutes	78.09 [32.30–96.33]	63.08 [31.16–86.41]	0.01	0.02^a^^,^^b^
Actinobacteria	10.35 [0.54–67.34]	10.70 [1.19–39.61]	0.64	0.80
Fusobacteria	0.72 [0.02–46.99]	3.93 [0.02–64.33]	0.00	0.01^a^^,^^c^
Bacteroidetes	1.72 [0.04–25.98]	6.13 [0.21–38.29]	0.00	0.01^a^^,^^c^
Proteobacteria	0.74 [0.04–4.62]	0.87 [0.05–16.49]	0.36	0.65
Campilobacterota	6.6e^–3^ [0–5.01]	0.16 [3.9e^–3^–4.27]	0.00	0.001^a^^,^^c^
Deinococcus-Thermus	0 [0–2.7e^–3^]	0 [0–1.7e^–3^]	0.55	0.80
Cyanobacteria/Chloroplast	0.01[0–0.92]	9.35e^–3^ [0–0.26]	0.75	0.80
Verrucomicrobia	0.04 [0–0.49]	7.75e^–3^ [0–0.12]	0.80	0.80
**Phylum**	**PLANT^1^ baseline**	**PLANT^1^ exit**	***p*-Value**	**FDR^3^**
Firmicutes	71.77 [34.17–91.02]	78.09 [32.30–96.33]	0.04	0.15
Actinobacteria	8.33 [0.6515–15.9701]	10.35 [0.54–67.34]	0.12	0.21
Fusobacteria	6.11 [0.2737–60.6256]	0.72 [0.02–46.97]	0.001	0.001^a^^,^^d^
Bacteroidetes	4.31 [0.23–20.60]	1.72 [0.04–25.98]	0.05	0.15
Proteobacteria	1.06 [0.44–36.22]	0.74 [0.04–4.62]	0.12	0.21
Campilobacterota	0.04 [0–4.58]	0.01 [0–5.01]	0.27	0.35
Deinococcus-Thermus	0 [0–0.01]	0 [0–2.7e^–3^]	0.41	0.46
Cyanobacteria/Chloroplast	0.01 [9e^–4^–0.483]	0.01 [0–0.92]	0.68	0.68
Verrucomicrobia	2.7e^–3^ [0–0.08]	0.04 [0–0.49]	0.19	0.29
**Phylum**	**MEAT^2^ baseline**	**MEAT^2^ exit**	***p*-Value**	**FDR^3^**
Firmicutes	70.31 [27.40–91.92]	63.08 [31.16–86.41]	0.40	0.72
Actinobacteria	6.06 [0.54–23.99]	10.70 [1.19–39.61]	0.09	0.71
Fusobacteria	6.73 [0.21–32.09]	3.93 [0.02–64.34]	0.62	0.72
Bacteroidetes	6.87 [0.14–39.08]	6.13 [0.21–38.29]	0.64	0.72
Proteobacteria	1.77 [0.02–15.45]	0.87 [0.05–16.49]	0.23	0.71
Campilobacterota	0.10 [5e^–4^–2.69]	0.16 [3.9e^–3^–4.27]	0.74	0.74
Deinococcus-Thermus	0 [0–0.01]	0 [0–1.7e^–3^]	0.47	0.72
Cyanobacteria/Chloroplast	6.2e^–3^[0–0.06]	0.01 [0–0.26]	0.52	0.72
Verrucomicrobia	2e^–3^ [0–0.07]	0.01 [0–0.12]	0.24	0.71

Comparisons were made between the baseline and exit time-points for each diet group. As data was presented as non-parametric alpha-diversity indices between diet group at each timepoint are presented as median and interquartile range [minimum and maximum].

^1^PLANT, plant-based diet.

^2^MEAT, animal-based diet.

^3^FDR, false discovery rate.

^a^Coefficient of correlation significant at *p <* 0.05.

^b^Denotes higher relative abundance in the PLANT group compared to the MEAT group.

^c^Denotes lower relative abundance in the PLANT group compared to the MEAT group.

^d^Denotes a decrease in relative abundance over time.

## Discussion

The present study revealed that feeding an entirely plant-based or vegan diet for 3 months had minor effects on the gut microbiota in dogs when compared to a conventional animal-based diet. A recent study conducted a gene catalog comparison between dog, human, pig, and mouse to determine which animal the dog’s GI microbiota was most closely related to (Coelho et al., 2018). The distribution of genes at the phylum level of this study showed that the canine GI microbiota has a higher taxonomic and functional overlap with the human GI microbiota (Coelho et al., 2018). These structural and functional similarities imply that human research findings could predict results in dogs and vice versa (Coelho et al., 2018). Based on this similarity to the human gut microbiota, it was expected that similar fecal microbiota changes consisting of increased abundance of microbiota that aid in carbohydrate fermentation and decreased abundance of microbiota related to fermentation of protein and fat would occur in dogs fed an entirely plant-based diet as those seen in humans consuming a vegan diet compared to an omnivorous diet. However, in the current canine study the bacterial fecal microbiota was minimally affected. Despite the lack of change in beta-diversity comparisons, there were a few taxonomic changes on the phylum level between the plant-based and animal-based diet groups, but no significant differences were found at the family or genus level.

In humans, a healthy adult intestinal microbiota is characterized by the dominance of Bacteroidetes and Firmicutes phyla (Simpson and Campbell, 2015; Bamberger et al., 2018). Variability between these two bacteria is shown to be heavily affected by diet, specifically between omnivorous, vegetarian, or vegan diets. In one study comparing Indian participants, whose diets mainly contained plant-based foods, with Chinese participants, whose diets contain mainly animal-based fats and proteins, researchers found the population of Bacteroidetes of Indian participants was nearly four times greater than in the Chinese individuals (Jain et al., 2018). An additional study involving European individuals identified a higher relative abundance of Bacteroidetes in vegans and vegetarians than in omnivores (Losasso et al., 2018). These findings agree with previous research showing that the relative abundance of Bacteroidetes in Italian children, typically consuming an omnivorous diet high in animal protein and fat, was less than half of that seen in Burkina Faso children who consume a diet rich in plant-based starch, fiber, and plant protein (De Filippo et al., 2010). These studies indicate that in humans a lower consumption of animal products is associated with a greater abundance of Bacteroidetes. In contrast, Lin et al. (2013) reported that children from the United States following a Western diet had a higher abundance of Bacteroidetes than children from Bangladesh following a Mediterranean diet. Like humans, the dominance of Bacteroidetes and Firmicutes was confirmed in healthy dogs (Deng and Swanson, 2015). However, the abundance of Bacteroidetes behaved differently in the present study in response to a plant-based diet. Dogs fed on the control animal-based diet had a higher abundance of Bacteroidetes than dogs on the experimental plant-based diet at the exit time-point. These distinct results in humans and dogs may be due to the many exogenous factors affecting the microbiota within the GI tract (Wu et al., 2011; Deng and Swanson, 2015; Li et al., 2017). For instance, results reported by Lin et al. (2013) indicated an abundance of Bacteroidetes between children consuming a Western diet compared to a Mediterranean diet; the researchers found both age and geographical differences as the potential explanation for their unexpected results (Lin et al., 2013). In dogs, similar significant associations between canine alpha-diversity indexes and geographical regions have been observed (Jha et al., 2020). Moreover, the same study observed differences in alpha-diversity indexes between rural and suburban dogs (Jha et al., 2020). In the current study, these exogenous factors may have played a role in affecting the GI microbiota, as client-owned dogs of various ages came from different household environments.

The balance between Bacteroidetes and Firmicutes is affected by each other where a decrease in Firmicutes levels usually occurs in favor of increasing Bacteroidetes levels (Tomova et al., 2019). The ratio between Bacteroidetes and Firmicutes in humans is related to a predisposition to disease development (Ley et al., 2006; Verdam et al., 2013). For example, increases in Firmicutes abundance are observed in obesity and GI diseases such as inflammatory bowel disease (Ley et al., 2006; Verdam et al., 2013). Humans consuming a vegan diet have a lower abundance of Firmicutes compared to omnivores; this finding is linked to positive health benefits, including weight loss, decreased inflammation, and improvements to the immune system that lower the risk of developing chronic diseases (Ley et al., 2006; Kim et al., 2013; Kumar et al., 2014; Singh et al., 2017). In the current study, however, dogs fed the experimental plant-based diet had a higher abundance of Firmicutes than the control animal-based diet. In human literature, these changes are attributed to endogenous factors that can affect the gut microbiota within the GI tract (Wu et al., 2011; Deng and Swanson, 2015; Li et al., 2017). In humans, the nutrient profiles between an omnivore diet and a plant-based diet are typically very different, with a vegan diet in humans expressing lower total energy and protein intake but increased fat and fiber intake when compared to an omnivorous diet (Clarys et al., 2014). In the current study, the experimental plant-based diet was formulated to have a similar nutrient profile to the control animal-based diet, despite different ingredients. Potentially, the changes in human fecal microbiota when comparing individuals consuming a vegan diet to individuals consuming an omnivorous diet may be in response to nutrient differences in the diets, not in response to ingredient changes. Thus, due to the nutrient profiles being as close as possible between the PLANT and MEAT diet this may be the reason that the canine gut microbiota in the current study was shown to be opposite of what is reported in human literature with dogs in the PLANT group expressing higher abundance of Firmicutes and lower abundance Bacteroidetes.

In humans, differences in Fusobacteria abundance have been documented when comparing individuals consuming a vegan diet to individuals consuming an omnivorous diet, with a greater abundance of Fusobacteria associated with an omnivorous diet (Losno et al., 2021). Similarly, the abundance of *Fusobacterium* (Fusobacteria phylum) was increased in dogs fed entirely animal-based raw food compared to dogs consuming omnivorous extruded diets (Pilla and Suchodolski, 2020). Fusobacteria is more abundant in other carnivorous species such as cats and wolves (Zhang and Chen, 2010; Bermingham et al., 2013). In the current study, relative abundance of Fusobacteria decreased from the baseline to exit time-point in dogs fed the PLANT diet. Furthermore, dogs fed the ANIMAL diet showed a higher abundance than those fed PLANT at the exit time-point. These results complement previous research found in humans (Losno et al., 2021) and other predator species (seals, tigers, polar bears, etc.) supporting that high abundance of Fusobacteria is associated with digestion of meat (Jiang et al., 2020).

The current study had several limitations including a smaller sample size, the use of client-owned animals, sample collection times, and the ability to extrapolate the study results to other vegan diets for dogs. Due to the relatively small sample size, one should be careful extrapolating of the results to a greater population. Nonetheless, the current study had a larger sample size then previously published gut microbiota research in client-owned dogs (Beloshapka et al., 2016; Herstad et al., 2017; Sanches et al., 2020; Onozawa et al., 2022). With use of client-owned animals we are reliant completely on their dedication to the trial protocol. Pet guardians were provided with a small Styrofoam cooler and waste collection bags and asked to collect feces immediately after defecation and freeze using the provided resources. With the use of client-owned animals, despite instructions to freeze fecal samples immediately after defecation, there was no supervision by the research team to ensure the fecal collection and storage was performed properly by pet guardians prior to delivery of the samples. However, the authors do not expect this to influence the results based on previous research in companion animals showing limited changes in the microbiota from short term (2 weeks) exposure to ambient temperature and time (Weese and Jalali, 2014; Tal et al., 2020; Chui et al., 2023). A further limitation factor with respect to data collection and the research variables assessed in the study was the frequency of sampling. Fecal collections from the dogs were only done at baseline (after the adaption period where all dogs were fed the MEAT diet) and exit (after feeding either the PLANT or MEAT diet exclusively for 3 months) timepoints. Based on recent research, the gut microbiota in dogs is very resilient meaning microbiota shifts and stabilization following a dietary intervention can be seen in just 2 weeks (Lin et al., 2022). In the current study changes may have occurred soon after introducing the MEAT or PLANT diet however, the pattern of change was indeterminable due to the data collection timepoints. This reduces the ability to interpret the significance of some of the changes. More frequent measurements to document the temporal trends and patterns in variables would have been beneficial. Furthermore, extrapolation of findings from this trial to all vegan dog foods should be made with caution. The PLANT diet used in the current trial was formulated to exceed minimum AAFCO nutrient recommendations (The Association of American Feed Control Officials, 2019) and also postproduction nutrient analysis occurred to ensure nutrient content was on target. Other commercially available vegan diets may not provide the same nutrient profile which could affect the gut microbiota composition. Lastly, COVID-19 lockdowns introduced some variability into the duration of the diet trial. Dogs were in various stages of the study (adaptation or experimental) when data collection was forcibly paused during the global pandemic. This limitation was unaccepted however, was taken into account during statistical analysis and the authors feel that the variation in trial duration did not influence the results.

## Conclusion

The current study showed that feeding an entirely plant-based or vegan diet for 12 weeks to healthy dogs has some potential to change the composition of the canine fecal microbiota, but these changes were not as dramatic or as distinct as those reported in humans. Future microbiota research investigating vegan dog food should consider the effects of different nutrient profiles as well as length of feeding. Moreover, further research is warranted to assess the metabolic function of the microbiota through fecal metabolomics and metagenomics in response to vegan dog foods, in addition to the fecal microbial composition and structural effects examined in the present study.

## Data availability statement

The datasets presented in this study can be found in online repositories. The names of the repository/repositories and accession number(s) can be found below: Borealis (the Universities Repository), https://doi.org/10.5683/SP3/VOELA6.

## Ethics statement

The animal studies were approved by the University of Guelph Animal Care Committee (AUP#4192) and the Research Ethics Board (Research Ethics Approval Number 19-02-036), and were in accordance with institutional, provincial, and national guidelines for the care and use of animals and humans participating in research. The studies were conducted in accordance with the local legislation and institutional requirements. Written informed consent was obtained from the owners for the participation of their animals in this study.

## Author contributions

BL: Writing – original draft. DG: Writing – review & editing. SD: Writing – review & editing. JM: Writing – review & editing. JA: Writing – review & editing. SB: Writing – review & editing. AV: Writing – review & editing.

## Supplementary material

Supplementary Figure 1 Flow chart of recruitment and enrollment of healthy adult client-owned dogs in a randomized, double-blinded longitudinal study investigation comparisons in fecal microbiota of an experimental plant-based (PLANT) versus a commercial animal-based (MEAT) extruded diet.
