# Supplementary material for: Comparison of the fecal microbiota of adult healthy dogs fed a plant-based (vegan) or an animal-based diet
Source: Front Microbiol. 2024 Apr 17;15:1367493. doi: 10.3389/fmicb.2024.1367493 (PMC11061427; doi:10.3389/fmicb.2024.1367493)
Supplement: Supplementary file 1 [file Table_1.docx]

**Table S1** Nutrient profile on dry matter basis and ingredient list of the experimental vegan (PLANT) and commercial animal-based (MEAT) extruded diets fed to the client-owned dogs in this randomized, double-blinded longitudinal study. Both diets were formulated to be isoenergetic, isonitrogenous, and as similar as possible in nutrient profiles.

| Nutrient, g/100g DM | PLANT^1^ | MEAT^2^ |
| --- | --- | --- |
| Moisture | 6.80 | 5.90 |
| Crude protein | 23.68 | 27.74 |
| Ether extract | 14.90 | 13.20 |
| Crude fibre | 3.90 | 3.40 |
| Crude ash | 7.10 | 8.10 |
| *Nitrogen free extract^3^ | 43.92 | 41.66 |
| **ME^4^, g/100kcal | 419 | 410 |
| PLANT Diet Ingredients | | |
| Peas, barley, oats, potato protein, sunflower oil (preserved with mixed tocopherols), pea protein, lentils, quinoa, calcium carbonate, dicalcium phosphate, primary dried yeast, flaxseed, natural vegetable flavouring, salt, dried marine algae, choline chloride, vitamins (vitamin A supplement, vitamin D2 supplement, vitamin E supplement, niacin, L-ascorbyl-2-polyphosphate (a source of vitamin C), d-calcium pantothenate, thiamine mononitrate, riboflavin, pyridoxine hydrochloride, folic acid, biotin, vitamin B12 supplement), minerals (zinc proteinate , iron proteinate, copper proteinate, zinc oxide, manganese proteinate, copper sulphate, ferrous sulphate, calcium iodate, manganous oxide, selenium yeast), DL-methionine, potassium chloride, L-lysine, taurine, L-carnitine, dried rosemary | | |
| MEAT Diet Ingredients | | |
| Chicken meal, de-boned chicken, whole brown rice, white rice, oatmeal, chicken fat (preserved with mixed tocopherols), potatoes, salmon meal, natural chicken flavour, whole dried egg, flaxseed, pea fibre, alfalfa, apples, carrots, cranberries, sodium chloride, potassium chloride, dried chicory root, dried Lactobacillus acidophilus fermentation product, dried Enterococcus faecium fermentation product, vitamins (vitamin A supplement, vitamin D3 supplement, vitamin E supplement, niacin, L-ascorbyl-2-polyphosphate (a source of vitamin C), d-calcium pantothenate, thiamine mononitrate, beta-carotene, riboflavin, pyridoxine hydrochloride, folic acid, biotin, vitamin B12 supplement), minerals (zinc proteinate, iron proteinate, copper proteinate, zinc oxide, manganese proteinate, copper sulphate, ferrous sulphate, calcium iodate, manganous oxide, selenium yeast), DL-methionine, L-lysine, taurine, yucca schidigera extract, dried rosemary. | | |

^1^PLANT, Plant-based diet
^2^MEAT, animal-based diet

^3^*NFE is calculated as: 100 - CP - EE - CF - CA (AAFCO, 2018).
^4^**ME (Kcal/kg) is calculated as: 10 × [(3.5 × CP) + (8.5 × EE) + (3.5 × NFE)] (AAFCO, 2019)
