# Supplementary material for: Comparison of the fecal microbiota of adult healthy dogs fed a plant-based (vegan) or an animal-based diet
Source: Front Microbiol. 2024 Apr 17;15:1367493. doi: 10.3389/fmicb.2024.1367493 (PMC11061427; doi:10.3389/fmicb.2024.1367493)
Supplement: Supplementary file 2 [file Table_2.docx]

Table S2. PERMANOVA tests to compare time and diet on Bray-Curtis distance to examine population structures at each taxonomic level in 47 healthy adult client-owned dogs fed an experimental plant-based (PLANT, n=27) or commercial animal-based (MEAT, n=20) extruded diet in a 12-week randomized, double-blinded longitudinal study.

| **Phylum** | **MeanSqs^1^** | **R2^2^** | **Pr(>F)^3^** |
| --- | --- | --- | --- |
| Time | 0.05 | 0.01 | 0.38 |
| Treatment | 0.14 | 0.02 | 0.53 |
| Time*Treatment | 0.03 | 0.01 | 0.54 |
| **Family** | **MeanSqs^1^** | **R2^2^** | **Pr(>F)^3^** |
| Time | 0.37 | 0.03 | 0.01^a^ |
| Treatment | 0.20 | 0.02 | 0.05 |
| Time*Treatment | 0.08 | 0.01 | 0.80 |
| **Genus** | **MeanSqs^1^** | **R2^2^** | **Pr(>F)^3^** |
| Time | 0.41 | 0.02 | 0.01^a^ |
| Treatment | 0.20 | 0.01 | 0.08 |
| Time*Treatment | 0.12 | 0.01 | 0.77 |

^1^MeanSqs, mean square
^2^R2, coefficient of determination
^3^Pr(>F), p-value
^a^Coefficient of correlation significant at P<0.05.
As Data was presented as non-parametric alpha-diversity indices between diet group at each timepoint are presented as median and interquartile range (minimum and maximum)
