# Supplementary material for: Comparison of the fecal microbiota of adult healthy dogs fed a plant-based (vegan) or an animal-based diet
Source: Front Microbiol. 2024 Apr 17;15:1367493. doi: 10.3389/fmicb.2024.1367493 (PMC11061427; doi:10.3389/fmicb.2024.1367493)
Supplement: Supplementary file 4 [file Table_4.docx]

## Table S4 Median relative abundance of predominant taxonomic classifications on the family level (>0.01%) of bacteria from the feces of 47 healthy adult client-owned dogs fed an experimental plant-based (PLANT, n=27) or commercial animal-based (MEAT, n=20) extruded diet in a 12-week randomized, double-blinded longitudinal study. Comparisons were made between diet groups at baseline and exit time-points and between time-points within each diet group.

| **Family** | **PLANT^1^ Baseline** | **MEAT^2^ Baseline** | **P-Value** | **FDR^3^** |
| --- | --- | --- | --- | --- |
| Lachnospiraceae | 21.51[10.85 – 51.19] | 20.78 [5.10 – 43.29] | 0.43 | 0.75 |
| Peptostreptococcaceae | 18.36 [0.48 – 41.25] | 15.04 [3.61 – 44.52] | 0.91 | 0.95 |
| Erysipelotrichaceae | 8.80 [0.52 – 23.67] | 10.58 [0.62 – 39.41] | 0.47 | 0.75 |
| Fusobacteriaceae | 8.44 [0.27 – 60.63] | 5.01 [0.21 – 32.09] | 0.54 | 0.75 |
| Coriobacteriaceae | 6.20 [0.26 – 16.54] | 4.83 [1.36 – 23.95] | 0.54 | 0.75 |
| Selenomonadaceae | 2.00 [0.06 – 23.23] | 2.99 [0.02 – 52.94] | 0.40 | 0.75 |
| Bacteroidaceae | 2.72 [0.12 – 27.95] | 1.59 [0.11 – 18.69] | 0.69 | 0.84 |
| Streptococcaceae | 0.15 [0.01 – 29.01] | 0.06 [0 – 8.22] | 0.28 | 0.67 |
| Prevotellaceae | 0.91 [0.02 – 14.97] | 4.31 [0.01 – 11.73] | 0.08 | 0.53 |
| Bifidobacteriaceae | 0.01 [0 – 20.57] | 0.01 [0 – 5.87] | 0.51 | 0.75 |
| Enterobacteriaceae | 0.96 [0 – 36.10] | 0.19 [0 – 4.43] | 0.07 | 0.53 |
| Veillonellaceae | 0.03 [0 – 5.67] | 0.01 [0 – 59.17] | 0.23 | 0.67 |
| Ruminococcaceae | 1.16 [0.03 – 9.30] | 1.95 [0.08 – 7.14] | 0.89 | 0.95 |
| Clostridiaceae_1 | 0.07 [0 - 14.34] | 0.29 [0 – 6.71] | 0.26 | 0.67 |
| Lactobacillaceae | 0.035[0 – 40.44] | 0.016 [0.01 – 5.94] | 0.12 | 0.67 |
| Eggerthellaceae | 0.43 [0 - 3.23] | 0.76 [0 – 5.52] | 0.30 | 0.67 |
| Helicobacteraceae | 0.04 [0 - 3.62] | 0.05 [0 – 4.58] | 0.97 | 0.97 |
| Succinivibrionaceae | 0.006 [0-0.87] | 0.02 [0 – 8.07] | 0.31 | 0.67 |
| Peptococcaceae_1 | 0.01 [0 – 3.13] | 0.14 [0 – 5.19] | 0.28 | 0.67 |
| Acidaminococcaceae | 0.21 [0 – 1.95] | 0.19 [0 – 3.44] | 0.96 | 0.97 |
| Stutterellaceae | 0.15 [0.01 – 0.85] | 0.14 [0 – 1.95] | 0.76 | 0.87 |
| Staphylococcaceae | 0.01 [0 – 1.15] | 0 [0 – 0.21] | 0.25 | 0.67 |
| Erysipelatoclostridiaceae | 0.01 [0 – 2.77] | 0.01 [0 – 1.10] | 0.04 | 0.43 |
| Atopobiaceae | 0 [0 – 0.33] | 0 [0 – 2.53] | 0.46 | 0.75 |
| Campylobacteraceae | 0 [0 – 1.02] | 0 [0 – 0.70] | 0.68 | 0.84 |
| Moraxellaceae | 0 [0 – 0.06] | 0 [0 – 0.10] | 0.48 | 0.75 |
| Enterococcaceae | 0.01 [0 – 0.79] | 0.01 [0 – 2.29] | 0.19 | 0.67 |
| Porphyromonadaceae | 0 [0 – 0.59] | 0 [0 – 0.65] | 0.48 | 0.75 |
| Neisseriaceae | 0 [0 – 0.01] | 0 [0 – 0] | 0.45 | 0.75 |
| Deinococcaceae | 0 [0 – 0.01] | 0 [0 – 0] | 0.72 | 0.85 |
| Muribaculaceae | 0 [0 – 0.57] | 0 [0 – 0.44] | 0.78 | 0.87 |
| Pseudomonadaceae | 0 [0 – 0.03] | 0 [0 – 0.03] | 0.23 | 0.67 |
| Actinomycetaceae | 0.01 [0 – 0.04] | 0 [0 – 0.01] | 0.04 | 0.43 |
| Streptophyta | 0.01 [0 – 0.06] | 0.01 [0 – 0.48] | 0.60 | 0.80 |
| Clostridiales_Incertae_Sedis_XIII | 0 [0 – 0.02] | 0 [0 – 0.61] | 0.27 | 0.67 |
| Corynebacteriaceae | 0.01 [0 – 0.12] | 0 [0 – 0.15] | 0.01 | 0.32 |
| Rhodoeacteraceae | 0 [0 – 0.01] | 0 [0 – 0.01] | 0.69 | 0.84 |
| Micrococcaceae | 0 [0 – 0.03] | 0[0 - 0.01] | 0.02 | 0.36 |
| Peptoniphilaceae | 0 [0 – 0.12] | 0 [0 – 0.04] | 0.17 | 0.67 |
| **Family** | **PLANT^1^ baseline** | **PLANT^1^ Exit** | **P-Value** | **FDR^3^** |
| Lachnospiraceae | 19.34 [4.87 – 37.42] | 18.93 [9.43 – 29.85] | 0.99 | 1.00 |
| Peptostreptococcaceae | 7.99 [0.24 – 35.63] | 12.09 [2.39 – 59.53] | 0.05 | 0.64 |
| Erysipelotrichaceae | 16.25 [0.60 – 38.32] | 12.01 [1.26 – 52.11] | 0.27 | 0.65 |
| Fusobacteriaceae | 1.19 [0.02 – 64.34] | 2.066 [0.02 – 46.97] | 0.86 | 0.91 |
| Coriobacteriaceae | 6.46 [0.93 – 13.81] | 6.92 [0.27 – 39.42] | 0.29 | 0.65 |
| Selenomonadaceae | 3.13 [0.10 – 55.34] | 1.95 [0.16 – 11.23] | 0.58 | 0.75 |
| Bacteroidaceae | 1.01 [0.01 – 36.53] | 0.68 [0.01 – 8.35] | 0.43 | 0.68 |
| Streptococcaceae | 0.84 [0.01 – 61.31] | 1.87 [0- 36.88] | 0.21 | 0.64 |
| Prevotellaceae | 1.18 [0 – 22.57] | 0.79 [0.01 – 29.17] | 0.84 | 0.91 |
| Bifidobacteriaceae | 0.10 [0 – 26.49] | 0.36 [ – 45.23] | 0.37 | 0.67 |
| Enterobacteriaceae | 0.20 [0 – 4.59] | 0.40 [ 0 – 15.72] | 0.17 | 0.64 |
| Veillonellaceae | 0.11 [0 – 37.22] | 0.05 [0 – 26.22] | 0.66 | 0.83 |
| Ruminococcaceae | 1.36 [0.07 – 8.83] | 0.51 [0.12 – 7.53] | 0.13 | 0.64 |
| Clostridiaceae_1 | 0.21 [0 – 9.02] | 0.74 [0.01 – 8.51] | 0.41 | 0.67 |
| Lactobacillaceae | 0.03 [0 – 12.17] | 0.22 [0 – 14.81] | 0.16 | 0.64 |
| Eggerthellaceae | 0.35 [0 – 3.61] | 0.92 [0 – 3.53] | 0.24 | 0.65 |
| Helicobacteraceae | 0.01 [0 – 4.66] | 0.02 [0 – 0.63] | 0.46 | 0.69 |
| Succinivibrionaceae | 0.01 [0 – 5.56] | 0 [0 – 0.60] | 0.57 | 0.75 |
| Peptococcaceae_1 | 0 [0 – 1.23] | 0.04 [0 – 3.36] | 0.40 | 0.67 |
| Acidaminococcaceae | 0.07 [0 – 1.91] | 0.03 [0 – 1.33] | 0.31 | 0.65 |
| Stutterellaceae | 0.13 [0 – 3.96] | 0.06 [0 – 0.27] | 0.08 | 0.64 |
| Staphylococcaceae | 0 [0 – 0] | 0.01 [0 – 0.62] | 0.15 | 0.64 |
| Erysipelatoclostridiaceae | 0 [0 – 0.49] | 0.01 [0 – 3.09] | 0.19 | 0.64 |
| Atopobiaceae | 0 [ 0 – 3.31] | 0 [0 – 1.69] | 0.82 | 0.91 |
| Campylobacteraceae | 0 [0 – 4.27] | 0 [0 – 0.86] | 0.55 | 0.75 |
| Moraxellaceae | 0 [0 – 0.10] | 0 [0 – 0.31] | 0.29 | 0.65 |
| Enterococcaceae | 0.01 [0 – 1.43] | 0.02 [0 – 0.11] | 0.12 | 0.64 |
| Porphyromonadaceae | 0 [0 – 0.25] | 0 [0 – 0.32] | 0.79 | 0.91 |
| Neisseriaceae | 0 [0 – 0] | 0 [0 – 0.03] | 0.56 | 0.75 |
| Deinococcaceae | 0 [0 – 0] | 0 [0 – 0] | 0.41 | 0.67 |
| Muribaculaceae | 0.01 [0 – 0.16] | 0 [0 – 0.35] | 0.21 | 0.64 |
| Pseudomonadaceae | 0 [0 – 0.06] | 0 [0 – 0.08] | 0.12 | 0.64 |
| Actinomycetaceae | 0 [0 – 0.03] | 0 [0 – 1.50] | 0.16 | 0.64 |
| Streptophyta | 0.01 [0 – 0.91] | 0.02 [0 – 0.26] | 0.32 | 0.65 |
| Clostridiales_Incertae_Sedis_XIII | 0 [0 – 0.08] | 0 [0 – 1.72] | 0.82 | 0.91 |
| Corynebacteriaceae | 0 [0 – 0.11] | 0.02 [0 – 0.89] | 0.17 | 0.64 |
| Rhodoeacteraceae | 0 [0 – 0.01] | 0 [0 – 0.08] | 0.74 | 0.90 |
| Micrococcaceae | 0 [0 – 0.01] | 0 [0 – 1.06] | 0.36 | 0.67 |
| Peptoniphilaceae | 0 [0 – 0.04] | 0 [0 – 0.24] | 1.00 | 1.00 |
| **Family** | **PLANT^1^ baseline** | **PLANT^1^ Exit** | **P-Value** | **FDR^3^** |
| Lachnospiraceae | 21.51 [10.85 – 51.19] | 19.35 [4.87 – 37.43] | 0.18 | 0.53 |
| Peptostreptococcaceae | 18.36 [0.48 – 41.25] | 7.99 [0.24 – 35.63] | 0.09 | 0.49 |
| Erysipelotrichaceae | 8.80 [0.52 – 23.67] | 16.25 [0.60 – 38.32] | 0.01 | 0.12 |
| Fusobacteriaceae | 8.44 [0.27 – 60.63] | 1.19 [0.02 – 64.34] | 0.00 | 0.12 |
| Coriobacteriaceae | 6.20 [0.26 – 16.54] | 6.46 [0.93 – 13.81] | 0.89 | 0.98 |
| Selenomonadaceae | 2.00 [0.06 – 23.23] | 3.13 [0.10 – 55.34] | 0.29 | 0.61 |
| Bacteroidaceae | 2.72 [0.12 – 27.95] | 1.01 [0.01 – 36.53] | 0.17 | 0.53 |
| Streptococcaceae | 0.15 [0 – 29.01] | 0.84 [0 – 61.31] | 0.12 | 0.53 |
| Prevotellaceae | 0.91 [0.01 – 14.97] | 1.18 [0 – 22.57] | 0.70 | 0.95 |
| Bifidobacteriaceae | 0.01 [0 – 20.57] | 0.10 [0 – 26.49] | 0.07 | 0.46 |
| Enterobacteriaceae | 0.96 [0 – 36.09] | 0.20 [0 – 4.59] | 0.05 | 0.46 |
| Veillonellaceae | 0.03 [0 – 5.67] | 0.11 [0 – 37.22] | 0.44 | 0.79 |
| Ruminococcaceae | 1.16 [0.03 – 9.30] | 1.36 [0.07 – 8.83] | 0.62 | 0.86 |
| Clostridiaceae_1 | 0.07[0-14.34] | 0.21 [0 – 9.02] | 0.20 | 0.53 |
| Lactobacillaceae | 0.04[0 – 40.44] | 0.03 [0 – 12.17] | 0.90 | 0.98 |
| Eggerthellaceae | 0.43[0-3.23] | 0.35 [0 – 3.61] | 0.57 | 0.82 |
| Helicobacteraceae | 0.04[0-3.62] | 0.01 [0 – 4.66] | 0.35 | 0.64 |
| Succinivibrionaceae | 0[0-0.89] | 0.01 [0 – 5.56] | 0.99 | 0.99 |
| Peptococcaceae_1 | 0.01 [0 – 3.13] | 0.01 [0 – 1.23] | 0.93 | 0.98 |
| Acidaminococcaceae | 0.21 [0 – 1.95] | 0.07 [0 – 1.91] | 0.93 | 0.98 |
| Stutterellaceae | 0.15 [0.01 – 0.85] | 0.13 [0 – 3.96] | 0.56 | 0.82 |
| Staphylococcaceae | 0.01 [0 – 1.15] | 0 [0 – 0.01] | 0.28 | 0.61 |
| Erysipelatoclostridiaceae | 0.01 [0 – 2.77] | 0 [0 – 0.49] | 0.07 | 0.46 |
| Atopobiaceae | 0 [0 – 0.33] | 0 [ 0 – 3.31] | 0.12 | 0.53 |
| Campylobacteraceae | 0 [0 – 1.01] | 0 [0 – 4.27] | 0.92 | 0.98 |
| Moraxellaceae | 0 [0 – 0.05] | 0 [0 – 0.10] | 0.53 | 0.82 |
| Enterococcaceae | 0.01 [0 – 0.79] | 0.01 [0 – 1.43] | 0.16 | 0.53 |
| Porphyromonadaceae | 0 [0 – 0.59] | 0 [0 – 0.25] | 0.92 | 0.98 |
| Neisseriaceae | 0 [0 – 0.02] | 0 [0 – 0] | 0.90 | 0.98 |
| Deinococcaceae | 0 [0 – 0.01] | 0 [0 – 0] | 0.52 | 0.82 |
| Muribaculaceae | 0 [0 – 0.57] | 0.01 [0 – 0.16] | 0.19 | 0.53 |
| Pseudomonadaceae | 0 [0 – 0.03] | 0 [0 – 0.06] | 0.30 | 0.61 |
| Actinomycetaceae | 0.01 [0 – 0.04] | 0 [0 – 0.03] | 0.34 | 0.64 |
| Streptophyta | 0.01 [0 – 0.06] | 0.01 [0 – 0.91] | 0.83 | 0.98 |
| Clostridiales_Incertae_Sedis_XIII | 0 [0 – 0.02] | [0 – 0.08] | 0.06 | 0.46 |
| Corynebacteriaceae | 0.01 [0 – 0.12] | 0.01 [0 – 0.11] | 0.99 | 0.99 |
| Rhodoeacteraceae | 0 [0 – 0.01] | 0 [0 – 0.01] | 0.19 | 0.53 |
| Micrococcaceae | 0 [0 – 0.03] | [0 – 0.01] | 0.22 | 0.55 |
| Peptoniphilaceae | 0 [0 – 0.12] | 0 [0 – 0.04] | 0.53 | 0.82 |
| **Family** | **MEAT^2^ Baseline** | **MEAT^2^ Exit** | **P-Value** | **FDR^3^** |
| Lachnospiraceae | 20.78 [5.10 – 43.29] | 18.94 [9.43 – 29.85] | 0.66 | 0.77 |
| Peptostreptococcaceae | 15.04 [3.61 – 44.52] | 12.09 [2.39 – 59.54] | 0.64 | 0.77 |
| Erysipelotrichaceae | 10.58 [0.62 – 39.41] | 12.01 [1.27 – 52.11] | 0.54 | 0.77 |
| Fusobacteriaceae | 5.01 [0.21 – 32.09] | 2.07 [0.02 – 46.97] | 0.01 | 0.13 |
| Coriobacteriaceae | 4.83 [1.36 – 23.95] | 6.92 [0.27 – 39.42] | 0.15 | 0.43 |
| Selenomonadaceae | 2.99 [0.02 – 52.94] | 1.95 [0.16 – 11.23] | 0.56 | 0.77 |
| Bacteroidaceae | 1.59 [0.11 – 18.69] | 0.68 [0.01 – 8.35] | 0.07 | 0.29 |
| Streptococcaceae | 0.06 [0 – 8.22] | 1.87 [0.01- 36.88] | 0.00 | 0.02^a^ |
| Prevotellaceae | 4.32 [0.01 – 11.73] | 0.79 [0.01 – 29.17] | 0.16 | 0.43 |
| Bifidobacteriaceae | 0.01 [0 – 5.87] | 0.36 [0.01 – 45.23] | 0.00 | 0.02 |
| Enterobacteriaceae | 0.19 [ – 4.43] | 0.40 [0 – 15.72] | 0.36 | 0.77 |
| Veillonellaceae | 0.01 [0 – 59.17] | 0.05 [0 – 26.22] | 0.50 | 0.77 |
| Ruminococcaceae | 1.95 [0.08 – 7.14] | 0.51 [0.12 – 7.53] | 0.44 | 0.77 |
| Clostridiaceae_1 | 0.29 [0 – 6.71] | 0.74 [0.01 – 8.51] | 0.66 | 0.77 |
| Lactobacillaceae | 0.02 [0.01 – 5.94] | 0.22 [0 – 14.81] | 0.05 | 0.26 |
| Eggerthellaceae | 0.76 [0 – 5.52] | 0.92 [0.01 – 3.53] | 0.90 | 0.91 |
| Helicobacteraceae | 0.05 [0 – 4.5] | 0.02 [0 – 0.63] | 0.85 | 0.91 |
| Succinivibrionaceae | 0.02 [0 – 8.07] | 0 [0 – 0.60] | 0.61 | 0.77 |
| Peptococcaceae_1 | 0.14 [0 – 5.19] | 0.04 [0 – 3.36] | 0.67 | 0.77 |
| Acidaminococcaceae | 0.19 [0 – 3.44] | 0.03 [0 – 1.33] | 0.67 | 0.77 |
| Stutterellaceae | 0.14 [0 – 1.95] | 0.06 [0 – 0.27] | 0.02 | 0.13 |
| Staphylococcaceae | 0 [0 – 0.21] | 0.01 [0 – 0.62] | 0.09 | 0.33 |
| Erysipelatoclostridiaceae | 0.01 [0 – 1.10] | 0.01 [0 – 3.10] | 0.13 | 0.43 |
| Atopobiaceae | 0 [0 – 2.53] | 0 [0 – 1.69] | 0.42 | 0.77 |
| Campylobacteraceae | 0 [0 – 0.70] | 0 [0 – 0.90] | 0.90 | 0.91 |
| Moraxellaceae | 0 [0 – 0.10] | 0 [0 – 0.31] | 0.37 | 0.77 |
| Enterococcaceae | 0.01 [0 – 2.29] | 0.02 [0 – 0.11] | 0.44 | 0.77 |
| Porphyromonadaceae | 0 [0 – 0.65] | 0 [0 – 0.32] | 0.40 | 0.77 |
| Neisseriaceae | 0 [0 – 0] | 0 [0 – 0.03] | 0.30 | 0.72 |
| Deinococcaceae | 0 [0 – 0] | 0 [0 – 0] | 0.59 | 0.77 |
| Muribaculaceae | 0 [0 – 0.45] | 0 [0 – 0.35] | 0.91 | 0.91 |
| Pseudomonadaceae | 0 [0 – 0.03] | 0 [0 – 0.08] | 0.09 | 0.33 |
| Actinomycetaceae | 0 [0 – 0.01] | 0 [0 – 1.50] | 0.02 | 0.13 |
| Streptophyta | 0 [0 – 0.48] | 0.02 [0 – 0.26] | 0.23 | 0.61 |
| Clostridiales_Incertae_Sedis_XIII | 0 [0 – 0.61] | 0 [0 – 1.72] | 0.64 | 0.77 |
| Corynebacteriaceae | 0 [0 – 0.15] | 0.02 [0 – 0.89] | 0.00 | 0.06 |
| Rhodoeacteraceae | 0 [0 – 0.01] | 0 [0 – 0.08] | 0.70 | 0.78 |
| Micrococcaceae | 0[0 - 0.07] | 0 [0 – 1.06] | 0.06 | 0.28 |
| Peptoniphilaceae | 0 [0 – 0.04] | 0 [0 – 0.23] | 0.63 | 0.77 |

^1^PLANT, Plant-based diet
^2^MEAT, animal-based diet
^3^FDR, false discovery rate
^a^Coefficient of correlation significant at P<0.05.
As Data was presented as non-parametric alpha-diversity indices between diet group at each timepoint are presented as median and interquartile range (minimum and maximum)
