# Supplementary material for: Comparison of the fecal microbiota of adult healthy dogs fed a plant-based (vegan) or an animal-based diet
Source: Front Microbiol. 2024 Apr 17;15:1367493. doi: 10.3389/fmicb.2024.1367493 (PMC11061427; doi:10.3389/fmicb.2024.1367493)
Supplement: Supplementary file 5 [file Table_5.docx]

## Table S5 Median relative abundance of predominant taxonomic classifications on the genus level (>0.001%) of bacteria from the feces of 47 healthy adult client-owned dogs fed an experimental plant-based (PLANT, n=27) or commercial animal-based (MEAT, n=20) extruded diet in a 12-week randomized, double-blinded longitudinal study. Comparisons were made between diet group at baseline and exit time-points and between time-points within each diet group.

| **Genus** | **PLANT^1^ Baseline** | **MEAT^2^ Baseline** | **P-Value** | **FDR^3^** |
| --- | --- | --- | --- | --- |
| Peptacetobacter | 0.16[0 – 0.41] | 0.14 [0.03 – 0.44] | 0.87 | 0.98 |
| Blautia | 0.09 [0.02 – 0.23] | 0.07 [0.01 – 0.22] | 0.60 | 0.89 |
| Fusobacterium | 0.08 [0 – 0.60] | 0.05 [0 – 0.30] | 0.60 | 0.89 |
| Collinsella | 0.06 [0 – 0.17] | 0.05 [0.01 – 0.24] | 0.54 | 0.89 |
| Megamonas | 0.02 [0 – 0.23] | 0.30 [0 – 0.53] | 0.40 | 0.89 |
| Streptococcus | 0 [0 – 0.29] | 0 [0 – 0.08] | 0.27 | 0.73 |
| Phocaeicola | 0.02 [0 – 0.18] | 0.01 [0 – 0.13] | 0.69 | 0.93 |
| Holdemanella | 0.02 [0 – 0.05] | 0.03 [0 – 0.20] | 0.10 | 0.73 |
| Catenibacterium | 0.01 [0 – 0.15] | 0.01 [0 – 0.07] | 0.61 | 0.89 |
| Bifidobacterium | 0 [0 – 0.21] | 0 [0 – 0.06] | 0.51 | 0.89 |
| Prevotella | 0 [0 – 0.15] | 0.01 [0 – 0.10] | 0.19 | 0.73 |
| Turicibacter | 0 [0 – 0.09] | 0.01 [0 – 0.08] | 0.91 | 0.98 |
| Megasphaera | 0 [0 – 0.05] | 0 [0 – 0.55] | 0.19 | 0.73 |
| Faecalibacillus | 0 [0 – 0.16] | 0.02 [0 – 0.10] | 0.23 | 0.73 |
| Escherichia/Shigella | 0 [0 – 0.25] | 0 [0 – 0.04] | 0.15 | 0.73 |
| Faecalibacterium | 0.01 [0 – 0.08] | 0 [0 – 0.06] | 0.91 | 0.98 |
| Faecalimonas | 0 [0 – 0.06] | 0 [0 – 0.03] | 0.23 | 0.73 |
| Allobaculum | 0.01 [0 – 0.07] | 0.01 [0 – 0.07] | 0.11 | 0.73 |
| Romboutsia | 0 [0 – 0.04] | 0 [0 – 0.03] | 0.55 | 0.89 |
| Clostridium_XVIII | 0 [0 – 0.07] | 0 [0– 0.02] | 0.79 | 0.96 |
| Prevotellamassilia | 0 [0 – 0.05] | 0 [0 – 0.04] | 0.11 | 0.73 |
| Bacteroides | 0 [0 – 0.07] | 0 [0 – 0.02] | 0.69 | 0.93 |
| Clostridium_sensu_stricto | 0 [0 – 0.02] | 0 [0 – 0.05] | 0.42 | 0.89 |
| Klebsiella | 0 [0 – 0.34] | 0 [0 – 0] | 0.01 | 0.35 |
| Slackia | 0 [0 – 0.02] | 0.01 [0 – 0.05] | 0.45 | 0.89 |
| Limosilactobacillus | 0 [0 – 0.40] | 0 [0 – 0] | 0.21 | 0.73 |
| Cetobacterium | 0 [0 – 0.02] | 0 [0 – 0] | 0.48 | 0.89 |
| Mediterraneibacter | 0 [0– 0.03] | 0 [0 – 0.02] | 0.43 | 0.89 |
| Helicobacter | 0 [0 – 0.04] | 0 [0 – 0.05] | 0.97 | 0.98 |
| Ligilactobacillus | 0 [0 – 0.02] | 0 [0– 0.02] | 0.30 | 0.75 |
| Peptococcus | 0 [0 – 0.03] | 0 [0 – 0.05] | 0.28 | 0.73 |
| Phascolarctobacterium | 0 [0 – 0.02] | 0 [0 – 0.03] | 0.96 | 0.98 |
| Anaerobiospirillum | 0 [0 – 0.08] | 0 [0 – 0.08] | 0.87 | 0.98 |
| Sarcina | 0 [0 – 0.12] | 0 [0 – 0.06] | 0.60 | 0.89 |
| Dialister | 0 [0 – 0] | 0 [0 – 0.05] | 0.81 | 0.96 |
| Anaerostipes | 0 [0 – 0.02] | 0 [0 – 0.03] | 0.74 | 0.94 |
| Adlercreutzia | 0 [0 – 0.18] | 0 [0 – 0.02] | 0.55 | 0.89 |
| Sutterella | 0 [0– 0] | 0 [0 – 0.02] | 0.76 | 0.94 |
| Ruminococcus2 | 0 [0 – 0.04] | 0 [0 – 0.02] | 0.13 | 0.73 |
| Lactobacillus | 0 [0 – 0] | 0 [0 – 0] | 0.98 | 0.98 |
| Staphylococcus | 0 [0 – 0.01] | 0 [0 – 0] | 0.26 | 0.73 |
| Erysipelatoclostridium | 0 [0 – 0.03] | 0 [0 – 0.01] | 0.04 | 0.55 |
| Campylobacter | 0 [0 – 0.01] | 0 [0 – 0] | 0.68 | 0.93 |
| Acinetobacter | 0 [0 – 0] | 0 [0 – 0] | 0.36 | 0.85 |
| Anaerotignum | 0 [0 – 0.02] | 0 [0 – 0.02] | 0.01 | 0.35 |
| Enterococcus | 0 [0 – 0] | 0 [0 – 0.02] | 0.16 | 0.73 |
| Paeniclostridium | 0 [0 – 0] | 0 [0 – 0.02] | 0.03 | 0.53 |
| Fournierella | 0 [0 – 0.02] | 0 [0 – 0] | 0.97 | 0.98 |
| Clostridium_XlVb | 0 [0 – 0.01] | 0 [0 -0.01] | 0.24 | 0.73 |
| Allisonella | 0 [0 – 0.01] | 0 [0 – 0.01] | 0.72 | 0.94 |
| **Genus** | **PLANT^1^ Exit** | **MEAT^2^ Exit** | **P-Value** | **FDR^3^** |
| Peptacetobacter | 0.07 [0– 0.24] | 0.11 [0 – 0.59] | 0.04 | 0.55 |
| Blautia | 0.06 [0– 0.18] | 0.05 [ 0– 0.10] | 0.52 | 0.80 |
| Fusobacterium | 0.10 [0– 0.45] | 0.02 [ 0– 0.42] | 0.93 | 0.94 |
| Collinsella | 0.06 [0.01 – 0.14] | 0.07 [0 – 0.39] | 0.29 | 0.68 |
| Megamonas | 0.03 [0 – 0.55] | 0.02 [0– 0.11] | 0.58 | 0.80 |
| Streptococcus | 0.01 [0– 0.61] | 0.02 [0 – 0.37] | 0.22 | 0.68 |
| Phocaeicola | 0.01 [0– 0.28] | 0 [ 0– 0.06] | 0.55 | 0.80 |
| Holdemanella | 0.02 [0– 0.11] | 0.01 [ 0– 0.20] | 0.09 | 0.68 |
| Catenibacterium | 0.04 [0– 0.32] | 0.01 [ 0 – 0.22] | 0.14 | 0.68 |
| Bifidobacterium | 0 [0 – 0.26] | 0 [ 0 – 0.45] | 0.37 | 0.76 |
| Prevotella | 0.01 [0– 0.21] | 0.01[ 0– 0.27] | 0.68 | 0.85 |
| Turicibacter | 0 [0 – 0.26] | 0.01 [ 0 – 0.07] | 0.58 | 0.80 |
| Megasphaera | 0 [0 – 0.37] | 0 [ 0 – 0.17] | 0.29 | 0.68 |
| Faecalibacillus | 0 [0 – 0.08] | 0 [ 0– 0.14] | 0.25 | 0.68 |
| Escherichia/Shigella | 0 [0 – 0.05] | 0 [ 0– 0.16 | 0.23 | 0.68 |
| Faecalibacterium | 0 [0 – 0.08] | 0 [ 0– 0.07] | 0.32 | 0.70 |
| Faecalimonas | 0 [0– 0.06] | 0 [0 – 0.06] | 0.91 | 0.94 |
| Allobaculum | 0 [0– 0.04] | 0 [0 – 0.05] | 0.76 | 0.91 |
| Romboutsia | 0 [0 – 0.11] | 0.01 [0 – 0.04] | 0.59 | 0.80 |
| Clostridium_XVIII | 0 [0 – 0.05] | 0 [0 – 0.14] | 0.75 | 0.91 |
| Prevotellamassilia | 0 [0 – 0.05] | 0 [0 – 0.03] | 0.91 | 0.94 |
| Bacteroides | 0 [0 –0.20] | 0 [ 0 – 0] | 0.45 | 0.76 |
| Clostridium_sensu_stricto | 0 [0 – 0.05] | 0.01 [ 0– 0.08] | 0.03 | 0.55 |
| Klebsiella | 0 [0 – 0] | 0 [ 0 – 0] | 0.29 | 0.68 |
| Slackia | 0 [0 – 0.02] | 0 [ 0– 0.03] | 0.29 | 0.68 |
| Limosilactobacillus | 0 [0 – 0.02] | 0 [ 0 – 0.01] | 0.20 | 0.68 |
| Cetobacterium | 0 [0 – 0.52] | 0 [ 0 – 0] | 0.87 | 0.94 |
| Mediterraneibacter | 0 [0– 0.02] | [ 0 – 0.01] | 0.86 | 0.94 |
| Helicobacter | 0 [0 – 0.05] | 0 [ 0 – 0.01] | 0.46 | 0.76 |
| Ligilactobacillus | 0 [0 – 0.03] | 0 [ 0– 0.09] | 0.11 | 0.68 |
| Peptococcus | 0 [0 – 0.01] | 0 [ 0 – 0.03] | 0.43 | 0.76 |
| Phascolarctobacterium | 0 [0 – 0.02] | 0 [ 0 – 0.01] | 0.40 | 0.76 |
| Anaerobiospirillum | 0 [0 – 0.06] | 0 [ 0 – 0.01] | 0.30 | 0.68 |
| Sarcina | 0 [0 – 0.03] | 0 [ 0 – 0] | 0.84 | 0.94 |
| Dialister | 0 [0 – 0.07] | 0 [ 0 – 0.09] | 0.41 | 0.76 |
| Anaerostipes | 0 [0 – 0.04] | 0 [ 0 – 0.05] | 0.98 | 0.98 |
| Adlercreutzia | 0 [0 – 0.01] | 0 [ 0 – 0.01] | 0.04 | 0.55 |
| Sutterella | 0 [0– 0.04] | 0 [ 0 – 0] | 0.12 | 0.68 |
| Ruminococcus2 | 0 [0 – 0.01] | 0 [ 0 – 0.01] | 0.43 | 0.76 |
| Lactobacillus | 0 [0 – 0.08] | 0 [0 – 0.05] | 0.59 | 0.80 |
| Staphylococcus | 0 [0 – 0] | 0[0 – 0} | 0.22 | 0.68 |
| Erysipelatoclostridium | 0 [0 – 0] | 0 [ 0 – 0.03] | 0.19 | 0.68 |
| Campylobacter | 0 [0 – 0.04] | 0 [ 0 – 0.01] | 0.55 | 0.80 |
| Acinetobacter | 0 [0 – 0] | 0 [ 0 – 0] | 0.13 | 0.68 |
| Anaerotignum | 0 [0 – 0.009528] | 0 [ 0 – 0.01] | 0.67 | 0.85 |
| Enterococcus | 0 [0 – 0.01] | 0 [0 – 0] | 0.17 | 0.68 |
| Paeniclostridium | 0 [0 – 0.01] | 0 [ 0 – 0.03] | 0.12 | 0.68 |
| Fournierella | 0 [0 – 0] | 0 [ 0 – 0] | 0.04 | 0.55 |
| Clostridium_XlVb | 0 [0 – 0] | 0 [ 0 – 0] | 0.81 | 0.94 |
| Allisonella | 0 [0 – 0.01] | 0 [ 0 – 0.02] | 0.65 | 0.85 |
| **Genus** | **PLANT^1^ baseline** | **PLANT^1^ Exit** | **P-Value** | **FDR^3^** |
| Peptacetobacter | 0.16 [0– 0.41] | 0.07 [0 – 0.24] | 0.01 | 0.16 |
| Blautia | 0.09 [0.02 – 0.23] | 0.06 [0 – 0.18] | 0.19 | 0.60 |
| Fusobacterium | 0.08 [0 – 0.60] | 0.10 [0 – 0.45] | 0.00 | 0.05 |
| Collinsella | 0.06 [0 – 0.17] | 0.06 [0.01 – 0.14] | 0.89 | 0.95 |
| Megamonas | 0.02 [0 – 0.23] | 0.03 [0 – 0.55] | 0.30 | 0.75 |
| Streptococcus | 0 [0 – 0.29] | 0.01 [0 – 0.61] | 0.12 | 0.52 |
| Phocaeicola | 0.02 [0– 0.18] | 0.01 [0 – 0.28] | 0.15 | 0.55 |
| Holdemanella | 0.02 [0– 0.05] | 0.02 [0 – 0.11] | 0.03 | 0.31 |
| Catenibacterium | 0.01 [0 – 0.15] | 0.04 [0– 0.32] | 0.03 | 0.31 |
| Bifidobacterium | 0 [0 – 0.21] | 0 [0– 0.26] | 0.06 | 0.38 |
| Prevotella | 0 [0 – 0.15] | 0.01 [0– 0.21] | 0.77 | 0.95 |
| Turicibacter | 0 [0– 0.09] | 0.01 [0– 0.26] | 0.38 | 0.82 |
| Megasphaera | 0 [0 – 0.05] | 0 [0 – 0.37] | 0.30 | 0.75 |
| Faecalibacillus | 0 [0 – 0.16] | 0 [0 – 0.08] | 0.75 | 0.95 |
| Escherichia/Shigella | 0 [0 – 0.25] | 0 [0 – 0.05] | 0.15 | 0.55 |
| Faecalibacterium | 0.01 [0– 0.08] | 0 [0 – 0.08] | 0.78 | 0.95 |
| Faecalimonas | 0.01 [0 – 0.06] | 0.01 [0 – 0.06] | 0.64 | 0.95 |
| Allobaculum | 0.01 [0 – 0.07 | 0 [0– 0.04] | 0.22 | 0.65 |
| Romboutsia | 0 [0– 0.04] | 0 [0 – 0.11] | 0.51 | 0.95 |
| Clostridium_XVIII | 0 [0– 0.07] | 0.01 [0 – 0.05] | 0.41 | 0.86 |
| Prevotellamassilia | 0 [0 – 0.05] | 0 [0 – 0.05] | 0.70 | 0.95 |
| Bacteroides | 0 [0– 0.07] | 0 [0 –0.20] | 0.11 | 0.52 |
| Clostridium_sensu_stricto | 0 [0 – 0.02] | 0 [0 – 0.05] | 0.04 | 0.32 |
| Klebsiella | 0 [0 – 0.34] | 0 [0 – 0] | 0.00 | 0.05 |
| Slackia | 0 [0 – 0.02] | 0 [0 – 0.02] | 0.37 | 0.82 |
| Limosilactobacillus | 0 [0 – 0.40] | 0 [0 – 0.02] | 0.68 | 0.95 |
| Cetobacterium | 0 [0 – 0.02] | 0 [0 – 0.52] | 0.61 | 0.95 |
| Mediterraneibacter | 0 [0 – 0.03] | 0 [0 – 0.02] | 0.60 | 0.95 |
| Helicobacter | 0 [0 – 0.04] | 0 [0 – 0.05] | 0.35 | 0.82 |
| Ligilactobacillus | 0 [0 – 0.02] | 0 [0 – 0.03] | 0.86 | 0.95 |
| Peptococcus | 0 [0 – 0.03] | 0 [0 – 0.01] | 0.93 | 0.95 |
| Phascolarctobacterium | 0 [0 – 0.02] | 0 [0 – 0.02] | 0.91 | 0.95 |
| Anaerobiospirillum | 0 [0 – 0.08] | 0 [0 – 0.06] | 0.94 | 0.95 |
| Sarcina | 0 [0 – 0.12] | 0 [0 – 0.03] | 0.57 | 0.95 |
| Dialister | 0 [0 – 0.01] | 0 [0 – 0.07] | 0.83 | 0.95 |
| Anaerostipes | 0 [0 – 0.02] | 0 [0 – 0.04] | 0.50 | 0.95 |
| Adlercreutzia | 0 [0 – 0.18] | 0 [0 – 0.01] | 0.88 | 0.95 |
| Sutterella | 0 [0 – 0.01] | 0 [0 – 0.04] | 0.72 | 0.95 |
| Ruminococcus2 | 0 [0 – 0.04] | 0 [0 – 0.01] | 0.15 | 0.55 |
| Lactobacillus | 0 [0 – 0] | 0 [0 – 0.08] | 0.64 | 0.95 |
| Staphylococcus | 0 [0 – 0.01] | 0 [0 – 0] | 0.49 | 0.95 |
| Erysipelatoclostridium | 0. [0 – 0.03] | 0 [0 – 0] | 0.07 | 0.39 |
| Campylobacter | 0 [0 – 0.01] | 0 [0 – 0.04] | 0.92 | 0.95 |
| Acinetobacter | 0 [0 – 0] | 0 [0 – 0] | 0.29 | 0.75 |
| Anaerotignum | 0 [0 – 0.02] | 0 [0 – 0.01] | 0.61 | 0.95 |
| Enterococcus | 0 [0 – 0.01] | 0 [0 – 0.01] | 0.19 | 0.60 |
| Paeniclostridium | 0 [0 – 0] | 0 [0 – 0.01] | 0.95 | 0.95 |
| Fournierella | 0 [0 – 0.02] | 0 [0 – 0] | 0.91 | 0.95 |
| Clostridium_XlVb | 0 [0 – 0.01] | 0 [0 – 0] | 0.04 | 0.32 |
| Allisonella | 0 [0 – 0.01] | 0 [0 – 0.01] | 0.70 | 0.95 |
| **Genus** | **MEAT^2^ Baseline** | **MEAT^2^ Exit** | **P-Value** | **FDR^3^** |
| Peptacetobacter | 0.14 [0.03 – 0.44] | 0.11 [0 – 0.59] | 0.53 | 0.75 |
| Blautia | 0.07 [0.01 – 0.22] | 0.05 [ 0 – 0.10] | 0.19 | 0.45 |
| Fusobacterium | 0.05 [0– 0.30] | 0.02 [ 0 – 0.42] | 0.01 | 0.18 |
| Collinsella | 0.05 [0.01 – 0.24] | 0.07[0– 0.39] | 0.15 | 0.39 |
| Megamonas | 0.30 [0 – 0.53] | 0.02 [0 – 0.11] | 0.56 | 0.76 |
| Streptococcus | 0 [0 – 0.08] | 0.02 [0 – 0.37] | 0.00 | 0.03^a^ |
| Phocaeicola | 0.01 [0 – 0.13] | 0.01 [ 0 – 0.06] | 0.08 | 0.22 |
| Holdemanella | 0.03 [0 – 0.19] | 0.01 [ 0 – 0.20] | 0.16 | 0.39 |
| Catenibacterium | 0.01 [0 – 0.07] | 0.01 [ 0 – 0.22] | 0.26 | 0.55 |
| Bifidobacterium | 0 [0 – 0.06] | 0 [ 0 – 0.45] | 0.00 | 0.03^a^ |
| Prevotella | 0.01 [0 – 0.10] | 0.01[ 0– 0.27] | 0.80 | 0.90 |
| Turicibacter | 0.01 [0 – 0.08] | 0.01 [ 0– 0.07] | 0.30 | 0.60 |
| Megasphaera | 0 [0 – 0.55] | 0 [ 0 – 0.17] | 0.42 | 0.63 |
| Faecalibacillus | 0.02 [0 – 0.10] | 0.01 [ 0 – 0.14] | 0.64 | 0.80 |
| Escherichia/Shigella | 0 [0– 0.04] | 0 [ 0 – 0.16] | 0.41 | 0.63 |
| Faecalibacterium | 0.01 [0 – 0.06] | 0 [ 0– 0.07] | 0.36 | 0.63 |
| Faecalimonas | 0 [0– 0.03] | 0.01 [0– 0.06] | 0.35 | 0.63 |
| Allobaculum | 0.01 [0 – 0.07] | 0 [0 – 0.05] | 0.02 | 0.18 |
| Romboutsia | 0 [0– 0.03] | 0.01 [0– 0.04] | 0.06 | 0.22 |
| Clostridium_XVIII | 0.0 [0– 0.02] | 0 [0 – 0.14] | 0.34 | 0.63 |
| Prevotellamassilia | 0 [0 – 0.04] | 0 [0 – 0.03] | 0.03 | 0.19 |
| Bacteroides | 0 [0 – 0.02] | 0 [ 0 – 0.01] | 0.05 | 0.22 |
| Clostridium_sensu_stricto | 0 [0 – 0.05] | 0.01 [ 0 – 0.08] | 0.05 | 0.22 |
| Klebsiella | 0 [0 – 0] | 0 [ 0 – 0] | 0.80 | 0.90 |
| Slackia | 0.01 [0 – 0.05] | 0 [ 0– 0.03] | 0.69 | 0.85 |
| Limosilactobacillus | 0 [0 – 0.01] | 0 [ 0 – 0.01] | 0.07 | 0.22 |
| Cetobacterium | 0 [0 – 0] | 0 [ 0 – 0] | 0.26 | 0.55 |
| Mediterraneibacter | 0 [0 – 0.02] | 0 [ 0 – 0.01] | 0.84 | 0.90 |
| Helicobacter | 0 [0 – 0.05] | 0 [ 0 – 0.01] | 0.85 | 0.90 |
| Ligilactobacillus | 0 [0– 0.02] | 0 [ 0 – 0.09] | 0.02 | 0.19 |
| Peptococcus | 0 [0 – 0.05] | 0 [ 0 – 0.03] | 0.64 | 0.80 |
| Phascolarctobacterium | 0 [0 – 0.03] | 0 [ 0 – 0.01] | 0.55 | 0.76 |
| Anaerobiospirillum | 0 [0 – 0.08] | 0 [ 0 – 0.01] | 0.62 | 0.80 |
| Sarcina | 0 [0 – 0.06] | 0 [ 0 – 0] | 0.83 | 0.90 |
| Dialister | 0 [0 – 0.05] | 0 [ 0 – 0.10] | 0.48 | 0.71 |
| Anaerostipes | 0 [0 – 0.03] | 0 [ 0 – 0.05] | 0.88 | 0.90 |
| Adlercreutzia | 0 [0 – 0.02] | 0 [ 0 – 0.01] | 0.21 | 0.48 |
| Sutterella | 0 [0 – 0.02] | 0 [ 0 – 0] | 0.03 | 0.19 |
| Ruminococcus2 | 0 [0 – 0.02] | 0 [ 0 – 0.01] | 0.03 | 0.19 |
| Lactobacillus | 0 [0 – 0] | 0 [0 – 0.05] | 0.38 | 0.63 |
| Staphylococcus | 0 [0 – 0] | 0 [ 0 – 0] | 0.08 | 0.22 |
| Erysipelatoclostridium | 0 [0 – 0.01] | 0 [ 0 – 0.03] | 0.13 | 0.37 |
| Campylobacter | 0 [0 – 0.01] | 0 [ 0 – 0.01] | 0.90 | 0.90 |
| Acinetobacter | 0 [0 – 0] | 0 [ 0 – 0] | 0.90 | 0.90 |
| Anaerotignum | 0 [0 – 0.02] | 0 [ 0 – 0.01] | 0.05 | 0.22 |
| Enterococcus | 0 [0 – 0.02] | 0 [ 0– 0] | 0.41 | 0.63 |
| Paeniclostridium | 0 [0 – 0.02] | 0 [ 0 – 0.03] | 0.00 | 0.05 |
| Fournierella | 0 [0 – 0] | 0 [ 0 – 0] | 0.07 | 0.22 |
| Clostridium_XlVb | 0 [0 -0.01] | 0 [ 0 – 0] | 0.42 | 0.63 |
| Allisonella | 0 [0 – 0.01] | 0 [ 0 – 0.02] | 0.71 | 0.85 |

^1^PLANT, Plant-based diet
^2^MEAT, animal-based diet
^3^FDR, false discovery rate
^a^Coefficient of correlation significant at P<0.05.
As Data was presented as non-parametric alpha-diversity indices between diet group at each timepoint are presented as median and interquartile range (minimum and maximum)
