# Supplementary figures and images for: Comparison of the fecal microbiota of adult healthy dogs fed a plant-based (vegan) or an animal-based diet
Source: Front Microbiol. 2024 Apr 17;15:1367493. doi: 10.3389/fmicb.2024.1367493 (PMC11061427; doi:10.3389/fmicb.2024.1367493)

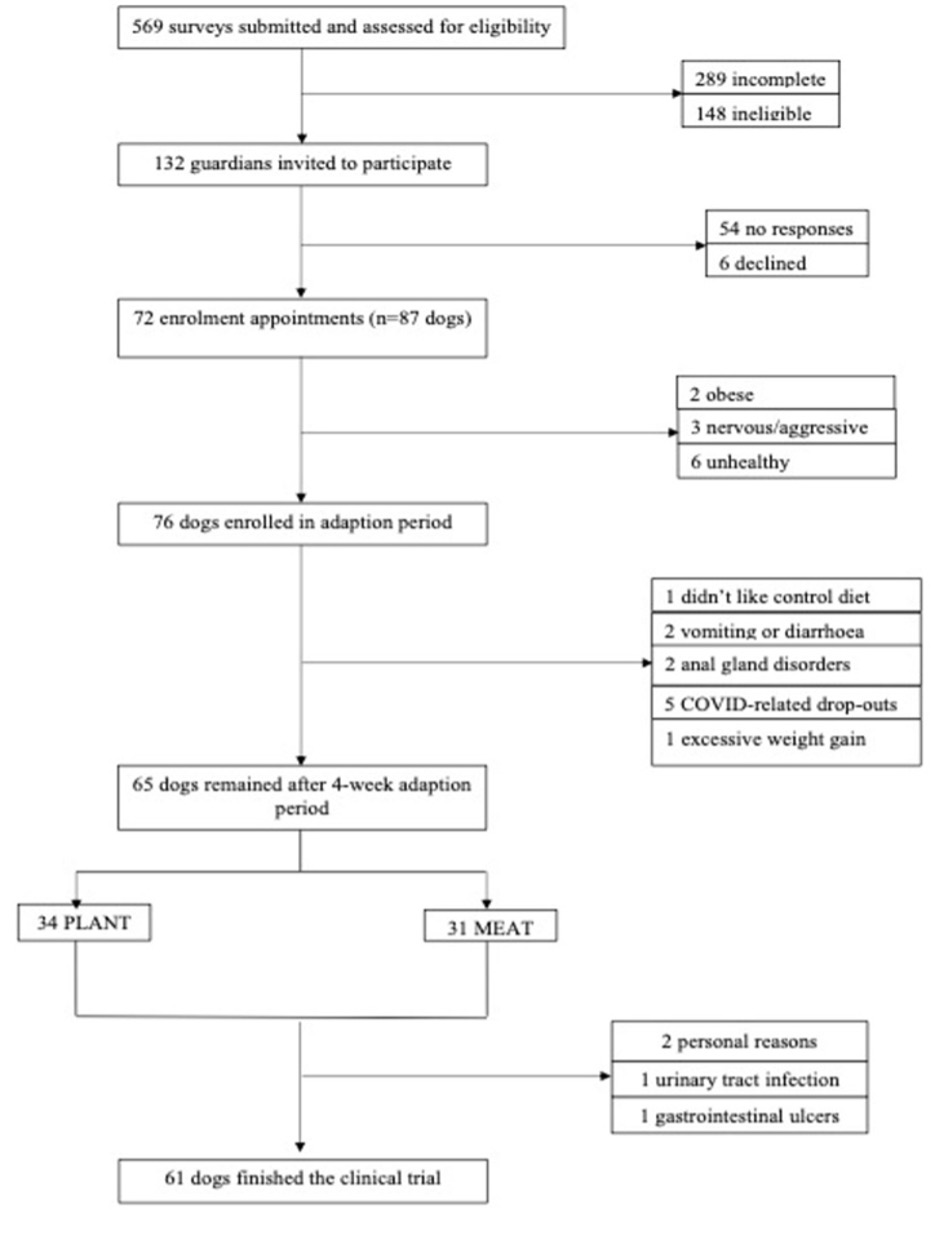

Supplement: Supplementary Figure 1 — Flow chart of recruitment and enrollment of healthy adult client-owned dogs in a randomized, double-blinded longitudinal study investigation comparisons in fecal microbiota of an experimental plant-based (PLANT) versus a commercial animal-based (MEAT) extruded diet. [file Image_1.JPEG]
